# A method for achieving high response rates in national surveys of U.S. primary care physicians

**DOI:** 10.1371/journal.pone.0202755

**Published:** 2018-08-23

**Authors:** Michaela Brtnikova, Lori A. Crane, Mandy A. Allison, Laura P. Hurley, Brenda L. Beaty, Allison Kempe

**Affiliations:** 1 Adult and Child Center for Health Outcomes Research and Delivery Science, University of Colorado Anschutz Medical Campus and Children’s Hospital Colorado, Aurora, CO, United States of America; 2 Department of Pediatrics, University of Colorado Anschutz Medical Campus and Children’s Hospital Colorado, Aurora, CO, United States of America; 3 Department of Community and Behavioral Health, Colorado School of Public Health, Denver, CO, United States of America; 4 Division of General Internal Medicine, Denver Health, Denver, CO, United States of America; University of Tennessee Health Science Center, UNITED STATES

## Abstract

Physician questionnaires are commonly used in health services research; however, many survey studies are limited by low response rate. We describe the effectiveness of a method to maximize survey response without using incentives, the effectiveness of survey reminders over time, and differences in response rates based on survey mode and primary care specialty. As part of a study to assess vaccine policy issues, 13 separate surveys were conducted by internet and mail over the period of 2008 to 2013. Surveys were conducted among pre-recruited networks of pediatricians, family physicians and general internists. Each network was active for 2 years and responded to 3–6 surveys. Physicians who indicated preference to respond through an online survey received up to 9 e-mailed requests to complete the questionnaire and up to 2 mailed questionnaires. Physicians who chose to respond by mail received up to 3 mailed questionnaires and a reminder postcard. For 6 of the 13 surveys conducted over the 6 year period, an additional mailing using a hand-addressed envelope was mailed to non-responders at the end of the usual protocol. Effectiveness of survey methods was measured by response rates. The overall response rates varied from 66‒83%. Response rates declined 17 percentage-points on average between the first and last surveys administered within each physician network. The internet group consistently had higher response rates than the mail group (74% vs. 62% on average). An additional mailing in a hand-written envelope boosted the final response rate by 11 percentage-points. Self-selection of survey mode, multiple reminders, and hand-written envelopes are effective methods for maximizing response rates in physician surveys.

## Introduction

Surveys are a useful and relatively inexpensive tool for examining physicians’ practices and attitudes. Many U.S. organizations, such as the Centers for Disease Control and Prevention (CDC), American Academy of Pediatrics (AAP), American Academy of Family Physicians (AAFP), American College of Physicians (ACP) and American Medical Association (AMA), have used data gained from physician surveys to improve delivery of health services and guide development of policies.[1–4]

The survey response rate is a commonly used indicator of the representativeness of collected data in a survey study. When response rates are high, the potential for differences between respondents and non-respondents are lower increasing the likelihood that survey results can be generalized to the population sampled, and reducing the likelihood of response bias.[5] Achieving a high response rate is also important for increasing efficiency and reducing cost of surveys implementation.

Survey researchers across a number of social science disciplines in the U.S. and abroad have witnessed a gradual decrease in survey participation over time.[6–10] and response rates are generally lower for physicians than for the general public surveys, mainly due to physicians’ demanding work schedules and increasing frequency of being approached for surveys.[7–9, 11] Some literature suggests that mail and telephone surveys have been more successful in gaining higher response rates in the physician population than faxed or internet-based surveys.[7, 12, 13] However, other literature has demonstrated the opposite.[14] Recent studies suggest that the mixed mode survey, combining internet and mail surveys, achieves the highest response rates in a physician population.[11, 13, 15] Beebe et al (2007) compared two mixed mode surveys and concluded that a mailed survey followed by internet follow-up achieved a higher response rate than an internet survey followed by a mailed reminder, but the web/mail method was faster.[16]

A number of strategies have been recommended to improve response rates to mail surveys, including personalizing the cover letter,[12] sending a pre-letter,[17] using certified mail,[18] and attaching a postal stamp instead of using metered envelopes.[12] However, using these strategies are not always effective.[19]

As survey demand has increased, researchers have come up with new ideas about how to incentivize respondents. Various incentives have been used to achieve higher response rates including providing a pen,[20] a prize draw,[21] lottery,[22] candy,[23] and money. Some literature shows higher response rates among physicians when using monetary incentives,[7, 9, 12, 24] while other literature shows limited benefit of monetary incentives.[17, 19] Incentives may not always be an option for researchers, especially with larger studies. Many national survey studies use the AMA Master File to obtain a nationally representative sample of physicians. However, response rates, as reported in the literature in the last five years, vary widely, from 6–70%.[25–28] When incentives are not provided, responses are generally ≤30%,[25, 26] while studies reporting response rates ≥50% generally have provided incentives of $20 or more.[27, 28]

Our study used pre-recruited sentinel networks of physicians that are representative of national physicians’ organizations, including the American Academy of Pediatrics, American Association of Family Practice and American College of Physicians. A previous study by our group[29] compared the pre-recruited sentinel networks to randomly selected samples from the AMA Master File and showed them to be generally comparable in each primary care specialty with respect to physician demographic characteristics, practice characteristics and responses to key survey questions. Our team has concluded that this method for constructing a sample is an efficient and representative approach for conducting policy-relevant national surveys of physicians.[29] The advantage of using networks is that smaller sample sizes are needed to get a given number of responses compared to a traditional approach. The sentinel method is especially advantageous because it uses the internet to contact respondents, greatly reducing survey costs by reducing the need for printing and postage.

This paper describes a method designed to maximize survey response in primary care physicians without using incentives and evaluates the overall effectiveness of the method as well as the effectiveness of survey reminders in multiple surveys conducted over a 6 year period of time. While this study does not have the benefit of randomization to compare methods, it shows consistent success in achieving high response rates in primary care physicians.

## Methods

The study was conducted by the Vaccine Policy Collaborative Initiative, a program for conducting rapid response physician surveys developed collaboratively with the Centers for Disease Control and Prevention (CDC). The Colorado Multiple Institutional Review Board, protocol # 04–0944, at the University of Colorado Denver approved this study as exempt research not requiring written informed consent.

### Population

Between 2008 and 2013, eight national networks of primary care physicians, with approximately 400 physicians in each network, were established for the purposes of this survey program (3 pediatric, 3 family practice, and 2 general internal medicine). Each network was created to be representative of the American Academy of Pediatrics, American Association of Family Practice or American College of Physicians based on the proportion of members by region of the country (Northeast, South, Midwest, West), practice setting (private, managed care, community/hospital based; not available for FM networks), and practice location (urban inner-city, urban non inner-city/suburban, rural). To recruit each physician network, a random sample from the relevant national physician organization was obtained, physicians were invited by mail to participate, and then a quota system was used to assure inclusion of physicians to match the national organization’s membership by region of the country, practice setting, and practice location. A complete description of the development of these networks has been previously published.[29] Power calculations established that 300 completed surveys would yield 80% power with a 5% Type I error rate to detect at least a 16 percentage point difference when comparing dichotomous variables between 2 groups of equal size. Assuming a 75% survey response rate, each network was therefore designed to have approximately 400 participants.

### Study setting

Thirteen 8-page questionnaires on various immunization delivery topics were administered to family medicine (FM) networks, pediatric (Peds) networks, and general internal medicine (GIM) networks. Specific topics are listed in the Table 1. Each network was active for about two years, participated in up to 6 questionnaires and then was replaced by a newly recruited network.

**Table 1:** Response rates by survey and specialty.

Survey Topic	Month survey initiated	Response rate
Measles, mumps, rubella, varicella	Oct-08	Peds: 76% (321/425)
		FM: 71% (299/424)
Vaccine risk communication	Feb-09	Peds: 88% (366/416)
		FM: 78% (330/423)
Influenza	Jul-09	Peds: 79% (330/416)
		FM: 70% (298/424)
		GIM: 78% (337/432)
Meningococcal	Dec-09	Peds: 88% (367/419)
		FM: 63% (268/423)
Follow-up influenza	Mar-10	Peds: 74% (305/414)
		FM: 68% (277/409)
		GIM: 65% (281/430)
Human papillomavirus	Jul-10	Peds: 82% (343/419)
		FM: 63% (266/423)
Rotavirus	Nov-10	Peds: 70% (289/410)
		FM: 61% (243/401)
Peds vaccine financing issues	Apr-11	Peds: 70% (291/413)
		FM: 68% (290/427)
Vaccine barcodes	Sep-11	Peds: 71% (288/408)
		FM: 66% (276/420)
		GIM: 61% (260/428)
Adult immunization	Mar-12	FM: 62% (255/409)
		GIM: 79% (352/443)
Vaccine schedule spreading out	Jun-12	Peds: 70% (282/405)
	Sep-12	FM: 62% (244/396)
Adult vaccine financing issues	Jul-13	FM: 59% (227/387)
		GIM: 72% (317/438)
Human Papillomavirus 2	Oct-13	Peds: 82% (364/442)
		FM: 56% (209/373)

At the time of recruitment, in an effort to increase participation, physicians were given a choice to be contacted for surveys via mail or email and were included in the mail or internet group based on their preference. Participants could change their preference any time during their network participation. For any single questionnaire, if a physician requested to change groups during implementation, for analysis purposes they were counted in their initial group for that questionnaire.

### Mail questionnaire administration

We used an approach that primarily followed the Dillman’s Tailored Design Method.[30] Physicians in the mail group received a pre-letter informing them about the upcoming questionnaire and the importance of the study. Five days later, mail participants were sent a personalized cover letter with a self-administered paper questionnaire and a pre-stamped return envelope, followed by a reminder postcard five days later. Each correspondence was on a Vaccine Policy Collaborative Initiative letterhead and signed by the principal investigator. Questionnaires consisted of 68 to 118 closed-ended questions that took 10–20 minutes to complete. Non-respondents received up to two additional questionnaires at 2-week intervals and responses were collected up to twelve weeks after the initial mailing. If the response rate was less than 50% after eight weeks, we added an additional mail questionnaire in a hand-addressed envelope of a different size and color than sent previously and a new cover letter explaining the importance of individual’s response for the validity of the research study. If the 12-week period contained a holiday or if an additional mailing was included, the collection time for returned questionnaires was extended to be at least six weeks after the final mailing.

### Internet questionnaire administration

The internet questionnaires were administered through a web-based survey company, Verint, Melville, New York.[31] Prior to the launch date, participants received an emailed pre-letter informing them about an upcoming questionnaire in three days. The questionnaire link was included as plain text in the body of a second email. Each participant was assigned an ID number with a unique survey link.

Non-respondents were sent reminders to complete the questionnaire every 3–4 days (up to six reminders) and then two final reminders at 1-week intervals, for a total span of 6 weeks. The time period of 6 weeks was selected to match the time period necessary to complete the mail protocol in the mail group. After 6 weeks, all non-respondents (including those with incorrect email addresses) received up to two mailed questionnaires at 2-week intervals with a cover letter similar to the mail group and given the option to correct their email address or to switch to the mail group for future surveys. Similar to the mail group, if the response rate after 8 weeks was less than 50%, an additional mailing in a hand-addressed envelope and a new cover letter were sent to all internet non-respondents.

### Analysis

Descriptive statistics and chi-squared tests were used to describe and compare response rates between primary care specialties and over time. Response rates were plotted by time, by questionnaire topic, survey mode (email vs. mail) and physician specialty.

The response rate attributable to specific survey administration methods, which was used as a measure of effectiveness, was calculated based on questionnaire return date recorded upon each internet questionnaire completion and mail questionnaire receipt date. Responses received by mail were attributed to a particular mailing if they were received in the timeframe of 5 days after that mailing and 5 days after the next mailing.

Nonresponse bias was assessed for each survey by comparing demographic characteristics of survey respondents to non-respondents. Table 2 shows the overall comparison across all surveys.

**Table 2:** Comparison of respondents and non-respondents.

Characteristic	Did not respond to all surveys (n = 2111, 55%) Column %	Responded to all surveys (n = 1740, 45%) Column %	p-value	Responded to none (n = 497, 13%) Column %	Responded at least to one survey (n = 3354, 87%) Column %	p-value
Specialty						
FM	36.7	28.7	< .0001	31.8	33.3	0.80
GIM	18.4	28.0		23.3	22.6	
Peds	45.0	43.3		44.9	44.1	
Male, %	52.1	51.2	0.57	59.6	50.5	0.0002
Provider Age						
Less than 40	19.3	17.3	0.01	14.3	19.0	0.002
40–49	32.0	28.7		35.9	29.7	
50–59	31.8	36.1		30.1	34.2	
Over 60	17.0	17.9		19.8	17.1	
Practice setting, %						
Private practice	76.9	77.5	0.12	79.3	76.9	0.49
Community/hospital based	19.2	17.5		16.9	18.7	
HMO or MCO	3.8	5.0		3.8	4.4	
Practice location, %						
Urban, Inner city	30.6	39.5	< .0001	33.8	34.7	0.04
Urban, non-inner/Suburban	52.7	43.3		52.7	47.8	
Rural	16.8	17.2		13.5	17.5	
Region of the Country, %						
Midwest	23.6	23.5	0.10	24.6	23.4	0.005
Northeast	20.6	22.4		23.5	21.1	
South	35.4	32.0		36.8	33.5	
West	20.4	22.2		15.1	22.1	
Providers in practice						
1–5	53.3	48.4	0.003	55.5	50.5	0.06
6 or more	46.7	51.6		44.5	49.5	

Abbreviations: Peds = pediatricians, FM = family medicine physicians, GIM = general internal medicine physicians, HMO = health maintenance organization, MCO = managed care organization

## Results

### Overall response rates

The overall response rates ranged from 66 to 83% and varied by survey topic and primary care specialty. Survey topics and response rates by specialty are included in Table 1. Peds consistently had higher response rates (70–88%) than GIM (61–80%) and FM (56–78%) (p<0.001).

#### Mail vs. email response rate

**Fig 1:**
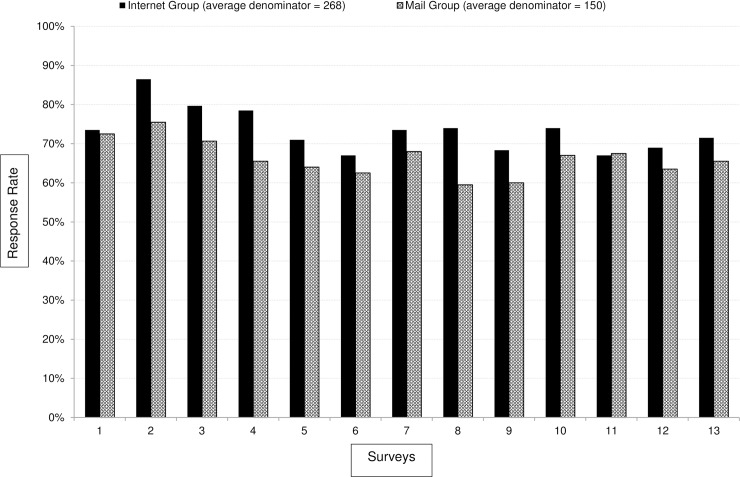
Internet vs. mail group response rates by survey (all specialties combines). Internet group received up to 9 emailed invitations/reminders and up to 2 mailed invitations/reminders. Mail group received up to 4 mailed invitations/reminders. Internet group had higher response rate than mail group with p<0.0001.

When offered to receive survey invitations by mail or email, on average, 53% of FM requested to be contacted by mail only, whereas only 30% of Peds and 28% of GIM preferred mail communication. In the majority of the 13 questionnaires, the email group’s survey response rates were higher than the mail group (Fig 1).

### Effectiveness of survey administration methods

**Fig 2:**
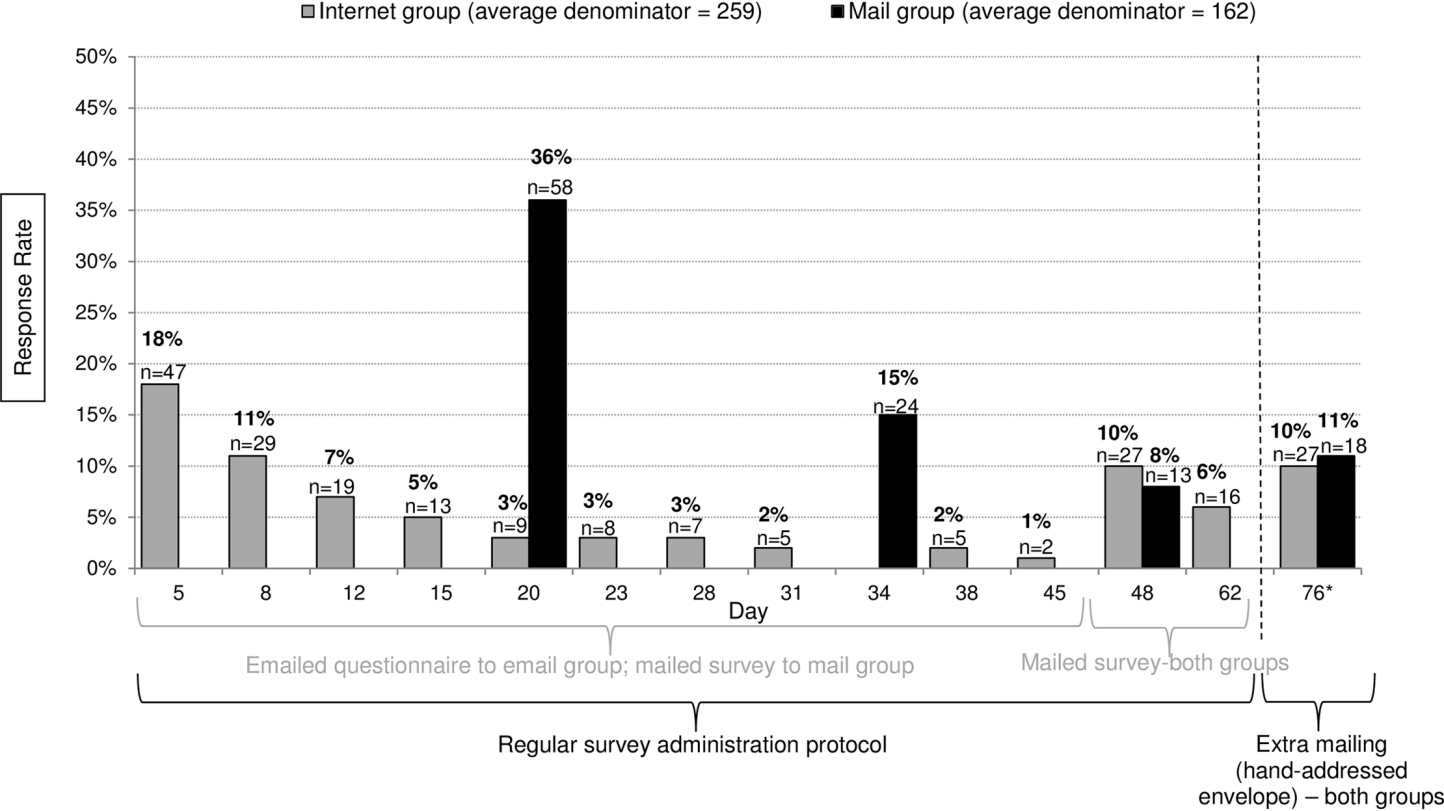
Internet vs. mail group average response rate by day (All specialties and surveys combined). In five survey studies, an additional hand-addressed envelope was sent to the internet group (average n = 259). In six survey studies an additional hand-addressed envelope was sent to the mail group (average n = 162).

The highest number of completed internet questionnaires was received between the first and second email invitations (Fig 2). The number of completed online questionnaires declined with every additional emailed survey reminder: while the first emailed survey had a nearly 20% response rate, the 8th survey reminder accomplished only an additional one percentage point of response. However, after 9 emails were sent and not responded to, mailed questionnaires added a substantial number of completed questionnaires (16 absolute percentage points in response rate from 2 mailings). When a final survey reminder was mailed in a hand-addressed envelope, an additional 15% of completed questionnaires was received (10 absolute percentage points in response rate). More physicians completed a questionnaire mailed in a hand-addressed envelope than a third reminder questionnaire mailed in the same envelope as the first and second questionnaires (Fig 2).

Fig 2 also shows the number of completed questionnaires per postal mailing. After the first mailed questionnaire, a response rate of 36% was recorded (on average 58 surveys); the second mailed questionnaire added 15 percentage points (24 returned surveys on average) and the third added only 8 percentage points (13 returned surveys). When an extra fourth mailing was sent in a hand-addressed envelope, an additional 11 percentage points were gained, which was on average 18 returned surveys (18% of all completed surveys). This was a higher response than the third mailing, which arrived in an envelope identical to the first mailed survey, without hand-addressing.

### Change in response rates over time within network

Regardless of when the network was initially recruited between 2008 and 2013, the response rates were similar. The mean response rate across all surveys administered to first Peds network was 78%, second Peds network 76%, first FM network 67%, second FM network 68% and third FM network 62%. For GIM the comparison was not possible because of the timeframe of this analysis.

#### Change in response rates over time between networks

**Fig 3:**
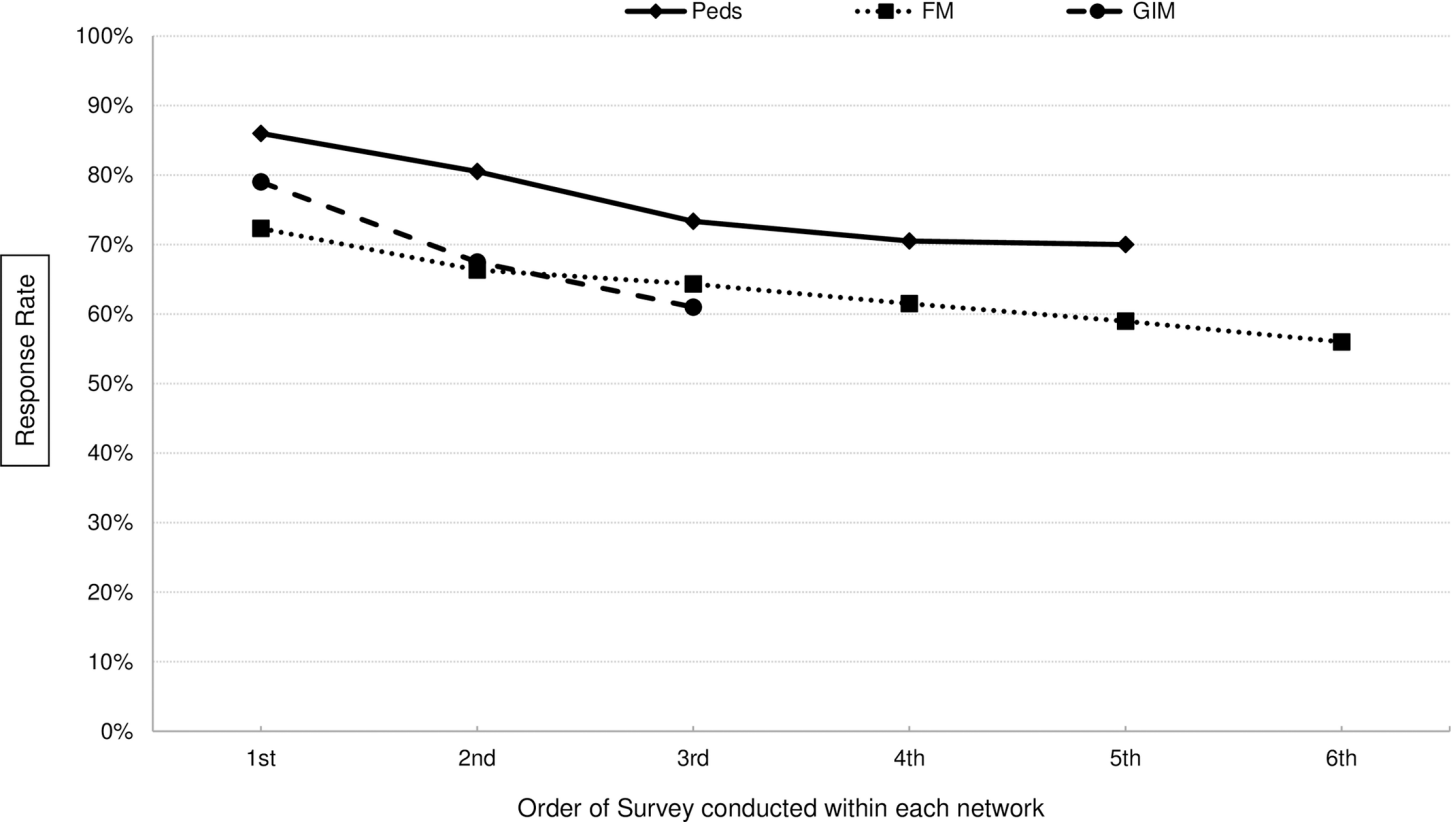
Decline in average response rates by the number of surveys administered. FM networks were invited to participate in the greatest number of surveys; whereas GIM networks were invited to participate in the least number of surveys.

The first survey administered to a newly recruited network consistently had the highest response rate, while the last survey administered to the same network had the lowest response rate. We saw declines up to 18 percentage points. The trend of declining response rates by specialty over time with each new survey administered is displayed in Fig 3.

## Discussion

This study was conducted to describe and evaluate a survey research method designed to achieve high response rates among U.S. primary care physicians. We found differences in response rates based on survey mode, primary care specialty, and effectiveness of various survey administration methods. The findings from our research can inform survey implementation procedures that increase efficiency in terms of both the time and cost.

Our mean overall response rate was higher than the majority of other national surveys of physicians.[25, 32] The highest response rates were achieved by pediatricians, perhaps because the survey topics dealt with immunization—traditionally an important part of pediatricians’ practice. The importance of topic relevance to survey response rates has been noted by Dillman et al.[30]

Our findings confirmed the results of previous research [11, 13, 30] regarding the effectiveness of mixed-mode surveys compared to mail-only surveys. The highest response rates were achieved in the email group when 9 attempts by email were followed by up to 3 attempts by mail, with the final mail attempt including additional features such as hand-addressing. Dillman [30] recommends that the final attempt have a different appearance than previous attempts, because “stimuli that are different from previous ones are generally more powerful than the repetition of a previously used technique.” Several other studies used this approach.[11] In our study, 6 out of 13 surveys had response rates lower than expected (<60%) after completing the 8 week survey administration protocol. The hand-addressed envelope was bigger in size and a different color than all previous mailings, and yielded in an additional 16–22% of completed questionnaires received (10–11 absolute percentage point increase in response rate). Our research showed that physicians were more likely to complete and return a 4th questionnaire mailed in an envelope of a different shape and color that was hand-addressed than a 3rd mail questionnaire in the same envelope as the first and second one (Fig 2). Perhaps more completed questionnaires would be returned if each additional mailed questionnaire was sent in a different size and/or color envelope, which has been previously suggested.[30]

While many newly recruited physician networks produced response rates over 80%, the response rates declined over time within network and two years later the same network produced on average a 17 percentage point lower response rate. This suggests that renewing physician networks more often or administering fewer surveys could accomplish higher response rates. It is demonstrated in our research (Fig 3) that after the first 4 administered surveys, response rates were still over 60% (over 70% in Peds) while after the 5^th^ and 6^th^ survey administered to the same network, the response rates declined to under 60%. Another solution to avoid declining response rates while maintaining the same number and length of surveys would be recruiting two networks for each specialty and alternating which network receives surveys.

Physician surveys can also benefit from knowing the email vs. mail preferences of primary care physicians in the U.S. Our research shows that about half of family physicians prefer to be contacted by mail only while less than one-third of general internists and pediatricians prefer mail over email. It is possible that this is influenced by practice location. More FM (26%) than GIM (15%) or Peds (11%) practice in rural areas[33–35] where the internet tends to be slower and used less [36] and more than 40% of FM used paper-based medical records at the time these surveys were conducted [37, 38], rather than computer-based systems. Conducting surveys by internet or mixed mode has obvious cost advantages due to lowering the need for printing and postage, and quicker turnaround time.

Our study has some possible limitations. Our methods for improving response rate were tested on pre-recruited physician networks[29] in which physicians had already agreed to complete surveys over a 2-year period. It is possible that the effectiveness of our survey methods would differ using a traditional approach in which the sample is not pre-recruited. It is possible that members of our pre-recruited samples were more interested in the topic of immunizations than those who declined participation in the networks, and that this had a generally positive effect on response rates. However, while overall response rates would most likely be lower, we expect that the pattern of results would be similar. Another potential limitation of our study is our ability to attribute survey responses to specific mailings. While each mail questionnaire was marked to distinguish between different mail campaigns, a later reminder may have initiated the physician to complete and return a questionnaire from an earlier mailing. Because the shortest time to return a completed questionnaire was 6 days after the study team mailed out the questionnaire, questionnaires received up to 5 days after the following mailing were assigned to the previous mailing. Thus, there may be some miss-assignment of responses if the postal delivery time was longer or shorter than expected.

Another limitation to our study could be the effect of survey topic on response rate. Some survey topics may have been more interesting to some physicians, resulting in higher response rates–yet our comparison treats all topics the same. Also, higher response rate in the email group could have been simply based on the higher number of reminders and not the mode itself. To address the lower response rates among physicians, researchers can implement some or all of our methods to increase the validity and cost-efficiency of their research.

In conclusion, our study over six years and 13 separate survey studies shows convincing evidence of the effectiveness of repeated invitations to participate and use of alternate modes of contact. It also shows that while overall survey response rates did not decline over the 6 year period, response rates did decline within groups of physicians who received invitations to participate in multiple survey studies. Finally, it suggests that there is an advantage to using internet surveys rather than mail surveys among respondents who indicate a preference for this method.

## Supporting information

S1 Data All data to replicate the study findings. (CSV)
